# An audit of request forms submitted in a multidisciplinary diagnostic center in Lagos

**DOI:** 10.11604/pamj.2015.20.423.5778

**Published:** 2015-04-29

**Authors:** Olufemi Abiola Oyedeji, Abiola Ann Ogbenna, Sandra Omozehio Iwuala

**Affiliations:** 1 Department of Haematology& Blood Transfusion, Faculty of Clinical Sciences, College of Medicine, University of Lagos, Nigeria; 2 Department of Medicine, Faculty of Clinical Sciences, College of Medicine, University of Lagos, Nigeria

**Keywords:** Audit, request forms, multidisciplinary diagnostic centre, Lagos

## Abstract

**Introduction:**

Request forms are important means of communication between physicians and diagnostic service providers. Pre-analytical errors account for over two thirds of errors encountered in diagnostic service provision. The importance of adequate completion of request forms is usually underestimated by physicians which may result in medical errors or delay in instituting appropriate treatment. The aim of this study was to audit the level of completion of request forms presented at a multidisciplinary diagnostic center.

**Methods:**

A review of all requests forms for investigations which included radiologic, laboratory and cardiac investigations received between July and December 2011 was performed to assess their level of completeness. The data was entered into a spreadsheet and analyzed.

**Results:**

Only 1.3% of the 7,841 request forms reviewed were fully completed. Patient's names, the referring physician's name and gender were the most completed information on the forms evaluated with 99.0%, 99.0% and 90.3% completion respectively. Patient's age was provided in 68.0%, request date in 88.2%, and clinical notes/ diagnosis in 65.9% of the requests. Patient's full address was provided in only 5.6% of requests evaluated.

**Conclusion:**

This study shows that investigation request forms are inadequately filled by physicians in our environment. Continuous medical education of physicians on the need for adequate completion of request forms is needed.

## Introduction

Request forms provide patient's details and information regarding the test to be performed and the importance of proper completion of these forms is usually emphasized early in physician training. Medical errors impact negatively on patient outcome [1] and modern medical practice is increasingly dependent on reliable clinical laboratory and radiological services [2]. Diagnostic errors may lead to increased costs and unnecessary deaths [3] and it has been demonstrated that laboratory results influence up to 70% of medical diagnoses [4]. Errors encountered in diagnostic service provision are usually divided into those in the pre-analytical, analytical and post-analytical phases of patient or sample testing. Majority (68.2%) of laboratory errors occur in the pre-analytical phase which refers to procedures performed before the sample or patient gets to the diagnostic service provider and are not under the control of the laboratory personnel e.g. completion of a laboratory request form, specimen collection and identification, phlebotomy, sample handling and transportation to the laboratory [5, 6]. The Royal College of Radiologists clearly suggests that all forms should be adequately and legibly completed to avoid any misunderstanding that may arise [7]. The clinician is required to state the reason for referral as this helps radiologists to better understand the patient's condition; so that the required expertise may be utilized to proffer the necessary information to aid proper patient management. Post-analytical error avoidance refers to the ultimate check on the pre-and intra-analytical quality. This includes the reviewing pathologist or radiologist providing interpretative comments and the clinician's interpretation and reaction to the results [5, 6]. The ability to correctly interpret results and the quality of interpretation given however is dependent on the quality of the information provided in the pre-analytical and analytical phases of testing [8]. The aim of this study is to assess the level of request form completion by physicians when requesting for investigations for their patients with a view to identify which information is most frequently overlooked.

## Methods

Request forms for investigations received at a comprehensive diagnostic center between July and December 2011 were retrospectively evaluated to measure the compliance of referring clinicians in adequate completion of request forms. The center is a privately owned tertiary level diagnostic center equipped to carry out routine and specialized radiologic, laboratory and cardiac investigations. Requests for investigations are received from clinicians practicing in primary, secondary and tertiary healthcare facilities in and around Lagos State, Nigeria. The requests evaluated in this study included all the investigative modalities offered at the center. Permission was obtained from the management of the center and ethical approval from the Health Research and Ethics Committee of the Lagos University Teaching Hospital. The information on request forms were entered into a Microsoft excel sheet and analyzed using Epi Info version 7.1.4.0 software developed by Centers for Disease Control and Prevention (CDC) in Atlanta, Georgia (USA). Each form was assessed for the presence and completeness of the information requested therein: demographic details; (full name, age, gender, full address of patients); the type of investigation required; a clinical history (defined as a clinical history and/or differential diagnosis); name and contact number of the requesting clinician; and request date. These data should be present on 100% of requests if completed correctly.

## Results

**Figure 1:**
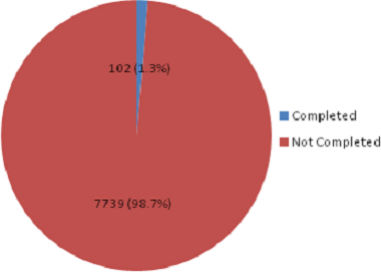
Completion status of the investigation request forms

There were 7925 request forms for investigations during the study period. Of these, 84 forms were excluded from the analysis as they were self-referrals. Thus, 7841 forms were included in the analyses. These forms included requests for laboratory, radiologic and cardiac investigations (Table 1). Of the 7841 forms evaluated, 7739 (98.7%) had one or more information missing (Figure 1). Patient's names, gender and the referring physician's name were the most completed information on the forms evaluated with 99.0%, 90.3% and 99.0% completion respectively. Patient's age was provided in 68.0%, request date in 88.2%, and clinical notes/ diagnosis in 65.9% of the requests. Patient's address was the least provided information with 5.6% completion in the forms evaluated (Table 2).

**Table 1:** Number of tests per modality on request forms

Type of investigation+		
Radiology/imaging	4465	56.9
Pathology (biochemistry, haematology, histology, immunology, microbiology)	3527	45.0
Cardiac/lung function	1031	13.1

+ Not mutually exclusive

**Table 2:** Completion rates of parameters on evaluated request forms

Information Required	Number completed N = 7841	Frequency (%)
**Patient's Name**		
Yes	7763	99.0
No	78	1.0
**Age**		
Yes	5330	68.0
Incomplete (e.g Adult)	1882	24.0
No	629	8.0
**Gender**		
Yes	7079	90.3
No	762	9.7
**Date of Request**		
Yes	6919	88.2
No	922	11.8
**Doctor's Name**		
Yes	7763	99.0
No	78	1.0
**Complete Address**		
Complete	439	5.6
Incomplete	210	2.7
None	7192	91.7
**Clinical notes/diagnosis**		
Yes	5170	65.9
No	2671	34.1

## Discussion

The importance of appropriate completion of investigation request forms is usually emphasized at orientation programs for newly employed doctors, especially pre-registration house officers. Investigative tests will be beneficial only if appropriate action is taken on the results obtained [9] thus every effort should be made to ensure proper information is provided when requesting for investigations to reduce pre-analytical errors. Studies from different parts of the world have however shown deficiencies in filling of laboratory [4, 8, 10, 11] or radiology request forms [7, 12, 13]. In this study, only 1.3% of requests were completely filled with the others having one or more parameters omitted. On the patient's biodata, patient's names had a 99.0% completion rate which is similar to findings by Olayemi and Asiamah-Broni [10], Burton and Stephenson [11] and Irurhe et al [13] who all reported a 100% completion but is higher than findings by Afolabi et al [12] who observed an 89.1% completion rate. Patient age was filled in 68.0% of requests although another 24.0% were filled indicating adult as "AD". Patient age has been reported to be filled in as low as 29.0% [8] to as high as 99.0% [11] although no study made mention of this inappropriate categorization. Patient gender was provided in 90.3% of requests which is comparable with other similar studies except by Olayemi and Asiamah-Broni [10] who reported 67.3% completion.

Even though the requests for investigations came from different clinicians and private hospitals, complete address was provided in only 5.6% of requests which is lower than reported in most other studies [7, 10–13]. Patient biodata and demographic details are of importance as they help in identification and results interpretation. Where patients have similar names, additional information is required to identify each patient or sort out their samples. Also several laboratory parameters have different reference ranges based on age and gender. These biodata also serve as a guide for radiologists to decide the appropriate radiological investigations and to limit patient exposure to unnecessary radiation which may be harmful [7]. The referring clinician's name and phone number were filled in 99.0% of forms which is higher than results reported in other studies [7, 10, 11]. Clinical notes/diagnosis where provided in 65.9% of forms evaluated. Providing relevant clinical information is important in proper interpretation of results and help in suggesting proper investigations to be carried out where the correct investigations were not initially requested. The high completion of referring doctor's names and phone numbers in this study is of benefit as calls could be placed to discuss errors in requests and relay urgent results that require immediate action to such clinicians. The date of request was provided in 88.2% of forms evaluated. This may not be relevant to the examination or reporting but becomes necessary when turn-around time is being considered or complaints about delays in reporting arise.

## Conclusion

This study shows that request forms for investigations are incompletely and inadequately filled. This will have an effect on the quality of service rendered as delays may arise while trying to obtain such omitted information, inappropriate tests may be performed on the patients or there may be difficulty in appropriate interpretation of obtained results. All these may have an effect on clinical decisions and management of patients. There is a need to continuously remind managing clinicians on the importance of adequately completing request forms for investigations. This can be achieved through continuous medical education programs where the importance of each parameter requested on the forms is further emphasized. A limitation of this study was our inability to assess the effect that this poor rate of completion had on turnaround time, interpretative comments and on patient's management.
